# Exploration and verification of circulating diagnostic biomarkers in osteoarthritis based on machine learning

**DOI:** 10.3389/fgene.2025.1513675

**Published:** 2025-02-17

**Authors:** Xinyu Wang, Tianyi Liu, Yueyang Sheng, Cheng Qiu, Yanzhuo Zhang, Yanqun Liu, Chengai Wu

**Affiliations:** ^1^ Department of Molecular Orthopaedics, National Center for Orthopaedics, Beijing Research Institute of Traumatology and Orthopaedics, Beijing Jishuitan Hospital, Capital Medical University, Beijing, China; ^2^ Department of Anaesthesia, National Center for Orthopaedics, Beijing Jishuitan Hospital, Capital Medical University, Beijing, China; ^3^ Department of Hepatobiliary Surgery, National Cancer Center/National Clinical Research Center for Cancer/Cancer Hospital, Chinese Academy of Medical Sciences and Peking Union Medical College, Beijing, China; ^4^ Department of Orthopedic Surgery, Peking Union Medical College Hospital, Peking Union Medical College and Chinese Academy of Medical Sciences, Beijing, China; ^5^ Trauma Reconstructive and Plastic Clinical Research Center, Yanbian University Hospital, Jilin, China

**Keywords:** osteoarthritis, biomarker, machine learning, SEH1L, BIRC2

## Abstract

**Background:**

Osteoarthritis (OA) is a prevalent chronic joint condition. This study sought to explore potential diagnostic biomarkers for OA and assess their relevance in clinical samples.

**Methods:**

We searched the GEO database for peripheral blood leukocytes expression profiles of OA patients as a training set to conduct differentially expressed gene (DEG) analysis. Two machine learning algorithms, least absolute shrinkage and selection operator (LASSO) logistic regression and support vector machine-recursive feature elimination (SVM-RFE), were employed to identify candidate biomarkers for OA diagnosis. The performance was assessed using receiver operating characteristic (ROC) curves, and the areas under the curve (AUCs) with 95% confidence interval (CI) were calculated. Furthermore, we gathered clinical peripheral blood samples from healthy donors and OA patients (validation set) to validate our findings. Small interfering RNA and CCK8 proliferation assay were used for experimental verification.

**Results:**

A total of 31 DEGs were discovered, and the machine learning screening found five DEGs that were considered to be candidate biomarkers. Notably, BIRC2 had a very good discriminatory effect among the five candidate biomarkers, with an AUC of 0.814 (95% CI: 0.697-0.915). In our validation set, results showed that the levels of BIRC2 and SEH1L were remarkably higher in healthy donors than OA patients, consistent with the results of the training set. SEH1L owned the largest AUC of 0.964 (95% CI: 0.855-1.000). BIRC2 also displayed a larger AUC of 0.836 (95% CI: 0.618-1.000) in the training set. Knockdown of these two genes could significantly suppress human chondrocyte proliferation.

**Conclusion:**

Two novel biomarkers, SEH1L and BIRC2, were indicated to have the capacity to differentiate healthy people from OA patients at the peripheral level. Experiments have shown that knockdown of these two genes could inhibit human chondrocyte proliferation, as verified by cell proliferation assays.

## Introduction

Osteoarthritis (OA) is a prevalent joint disorder, particularly among older adults, affecting more than 500 million people globally (Hunter et al., 2020). It is marked by the deterioration of cartilage, inflammation of the synovial membrane, changes in the subchondral bone, and the formation of osteophytes, ultimately leading to a decline in joint function (Murphy et al., 2019). As a result, OA is recognized as a significant contributor to global disability. Currently, OA is typically diagnosed based on clinical symptoms and imaging techniques, making early and accurate diagnosis challenging. This delay in diagnosis often allows the disease to progress in many patients, resulting in a poor outlook and limited treatment options (Deng et al., 2020).

Currently, there are no approved drugs that modify the progression of OA (Win Min, 2022; Pisetsky, 2021). Recent treatment guidelines from leading international organizations emphasize education, structured exercise, and weight loss as fundamental therapies (Bruyère et al., 2016; Bruyère et al., 2019; Kolasinski et al., 2019). They recommend topical non-steroidal anti-inflammatory drugs (NSAIDs) as the first line treatment, with oral NSAIDs and intra-articular injections reserved for managing ongoing pain (Bruyère et al., 2019; Kolasinski et al., 2019). Thus, treatment options for OA are mainly limited to prescribing pain relief medications, encouraging exercise, physical therapy, and, in some cases, surgical intervention (Bennell et al., 2015). The primary aim of treatment is to alleviate symptoms, enhance joint function, and prevent further joint damage, while the long-term goal of achieving true disease modification remains yet to be realized. While both pharmacological and non-pharmacological treatments are typically successful in reducing pain and enhancing physical function, they do not fundamentally alter the underlying pathological and radiographic changes associated with OA (Gelber, 2024). As the condition worsens, both pain and functional limitations tend to escalate. Therefore, identifying biomarkers for early diagnosis is essential for enhancing the prognosis of OA patients.

Biomarkers play a crucial role in diagnosing diseases and developing drugs. According to the European Medicines Agency, a biomarker is a biological molecule present in blood, other bodily fluids, or tissues that can be utilized to monitor bodily processes and diseases in both humans and animals. Currently, several clinical biomarkers have been identified in the context of OA, including cartilage oligomeric matrix protein (COMP), C-reactive protein (CRP), and matrix metalloproteinases (MMPs). COMP has been recognized for its role in cartilage metabolism and is often elevated in the serum of OA patients, while CRP serves as a general marker of inflammation that may correlate with disease severity. Additionally, MMPs, which are involved in extracellular matrix degradation, provide insights into the pathological processes underlying OA. However, these biomarkers have limitations in specificity and sensitivity, underscoring the need for the identification of novel biomarkers that can enhance diagnostic accuracy and therapeutic monitoring.

To discover and validate new biomarkers for OA, researchers commonly employ a variety of analytical methods. Transcriptomic and proteomic approaches, including microarray analysis and mass spectrometry, allow for the comprehensive profiling of gene and protein expression changes associated with OA. Furthermore, bioinformatics facilitates the integration of multi-omics data, enabling the identification of key regulatory networks and potential biomarker candidates. Additionally, machine learning algorithms are increasingly being utilized to analyze complex datasets, improving the predictive power of biomarker discovery. There is a growing focus on the early detection of OA, as it would enable more effective treatment options. This creates a “window of opportunity” for utilizing biomarkers to diagnose the disease sooner, potentially enhancing the clinical outcomes of existing treatments, despite their limitations. Nevertheless, employing biomarkers, particularly biochemical markers, during the asymptomatic phases of the disease proves to be quite difficult. Identifying biomarkers that possess the necessary sensitivity and specificity for this purpose remains a significant challenge in OA research.

In this study, we utilized two machine learning algorithms, least absolute shrinkage and selection operator (LASSO) logistic regression and support vector machine-recursive feature elimination (SVM-RFE), to identify biomarkers for predicting knee OA diagnosis. We began by extracting OA microarray datasets from the Gene Expression Omnibus (GEO) database, conducting differential expression analysis, and performing protein-protein interaction (PPI) analysis on the differentially expressed genes (DEGs) before applying machine learning techniques. To assess the accuracy and reliability of the candidate biomarkers, we plotted receiver operating characteristic (ROC) curves and calculated the areas under the curve (AUCs) with a 95% confidence interval (CI). Additionally, we collected clinical peripheral blood samples from healthy donors and knee OA patients to validate our findings. Small interfering RNA (siRNA), quantitative real-time PCR and CCK8 proliferation assay were used for experimental verification. We hope that biomarkers identified in our study could facilitate early diagnosis and improve prognosis of knee OA.

## Material and methods

### Data processing

The public database GEO (https://www.ncbi.nlm.nih.gov/geo/) (Barrett et al., 2013; Edgar et al., 2002) was mined in our study. The peripheral blood leukocytes expression profiles, along with the clinical information from the microarray expression dataset GSE63359, consisting of 26 healthy donors (healthy control, HC) and 45 knee osteoarthritis patients (OA), were downloaded. The GSE63359 dataset was annotated by the GPL96 platform. Data were normalized using the “normalizeBetweenArrays” of the “limma” package for further analysis. The average expression value was calculated for OA patient number 48 since the patient had two duplicate samples tested. R software (version 4.2.2) was utilized during this process, and “GEOquery” (Davis and Meltzer, 2007), “stringr”, “limma” (Ritchie et al., 2015), and “dplyr” packages were used for data processing. The GSE63359 dataset served as the training set in this study.

### DEG analysis and candidate diagnostic biomarker exploration

DEG analysis was conducted between healthy control and OA patients using the “limma” package. Genes meeting the following criteria: |log_2_ fold change (FC)| > 0.5 and *P*-value <0.05 were defined as DEGs, which were then subjected to protein-protein interaction analysis using the online STRING tool (version 12.0) (https://cn.string-db.org/) (Szklarczyk et al., 2023) where a medium confidence interaction score (0.400) was applied and disconnected nodes in the network were hidden. The “ggplot2” package was used to draw a volcano plot displaying the DEGs, and the top five upregulated and downregulated genes with the lowest *P*-value were labeled. Then the LASSO logistic regression, and SVM-RFE algorithms were employed to explore candidate circulating biomarkers for OA. LASSO regression was performed by the “glmnet” package and a ten-fold cross validation was applied. The genes corresponding to 1se.lambda were taken as the potential biomarkers. The SVM-RFE algorithm was conducted by the “e1071”, “caret” and “kernlab” (Karatzoglou et al., 2004) packages, with a ten-fold cross validation applied. For every cross validation, the average RMSE (Root Mean Squared Error) was recorded for each featured gene set. The featured gene set with the lowest RMSE was chosen as the candidate biomarker set. The filtered genes by either LASSO regression or SVM-RFE were considered as candidate biomarkers. ROC curves for each biomarker were drawn using the “pROC” package and AUCs as well as 95% CI were calculated. The expression level of each biomarker in both healthy donors and OA patients was shown by boxplots.

### Sample collection

The participants in this study were OA patients who were waiting for artificial joint replacement at Jishuitan Hospital. The inclusion criteria were: (1) diagnosis of OA in line with the standards set by the Orthopaedic Branch of the Chinese Medical Association; and (2) patients not currently receiving treatment with nonsteroidal anti-inflammatory drugs (NSAIDs). The exclusion criteria included: (1) individuals with a history of severe infections, surgeries, or tumors; (2) those with significant cardiac, pulmonary, liver, or kidney dysfunction; and (3) individuals with a history of autoimmune diseases such as rheumatoid arthritis or ankylosing spondylitis. Additionally, a control group was included, consisting of five healthy volunteers aged 49 to 62 who had not received any medical treatment in the past 6 months and had no other chronic conditions (like coronary artery disease, diabetes, or hypertension) or osteoarticular diseases (including skeletal fluorosis, gout, or rheumatoid arthritis). All participants were of Chinese Han descent. Written informed consent was obtained from all participants. Our study adhered to the Declaration of Helsinki and received approval from the Ethics Committee of Beijing Jishuitan Hospital (ethics code: 201611-03).

### RNA-sequencing assay

All collected blood samples were processed using the HiSeq platform with an Illumina TruseqTM RNA sample preparation kit. After constructing the library, SeqPrep (https://github.com/jstjohn/SeqPrep) was utilized to analyze the raw data and obtain qualified mRNA and lncRNA sequences for further analysis. The candidate biomarkers identified from the training set were validated using our own transcriptome sequencing data, which acted as the validation set.

### Cell isolation and culture

Human primary chondrocytes together with the culture system were bought from Beijing Beina Chuanglian Institute of Biotechnology. Cells were cultured in Dulbecco’s modified Eagle’s medium (DMEM)/F12 supplemented with 10% fetal bovine serum and 1% penicillin-streptomycin in a 37°C incubator under condition of 5% CO_2_. After reaching 80%–90% confluence, cells were digested with trypsin (Gibco, United States) and passaged twice per week at a split ratio of 1:3. Cells at passages 3-4 were used for the subsequent experiments.

### Cell transfection

The siRNAs targeting the *BIRC2* and *SEH1L*, as well as their corresponding negative controls, were purchased from RiboBio (Guangzhou, China) and transfected into human chondrocytes using Lipofectamine RNAiMAX transfection reagent (Invitrogen, United States) according to the manufacturer’s instructions with addition of Opti-MEM medium (Invitrogen, United States). The efficiency of gene knockdown was validated by qPCR experiments.

### Quantitative real-time PCR

Six-well plates were inoculated with chondrocytes, which were then cultured in a complete media. As directed by the manufacturer, total RNA was extracted from chondrocytes with the MiniBEST Universal RNA Extraction Kit (TaKaRa, Japan). To create cDNA and prevent DNA contamination, the PrimeScript™ RT reagent kit with gDNA Eraser (TaKaRa, Japan) was used. A TB Green Premix Ex Taq (TaKaRa, Japan) along with an Applied Biosystems 7,500 Real-Time PCR System was employed for real-time PCR in accordance with the manufacturers’ instructions. The comparative Ct (2^−ΔΔCT^) approach was used to examine the data and determine the relative gene expression. Table 1 includes a list of primer sequences.

**TABLE 1:** The primers for qRT-PCR.

Target	Sequence (5′-3′)
BIRC2-F	AGC​ACG​ATC​TTG​TCA​GAT​TGG
BIRC2-R	GGC​GGG​GAA​AGT​TGA​ATA​TGT​A
SEH1L-F	AGC​ACA​TGG​GTC​TTA​TGT​TAG​C
SEH1L-R	GAA​TGA​GCA​CGA​GAG​CTT​GAA
GAPDH-F	AAG​GGT​CAT​CAT​CTC​TGC​CC
GAPDH-R	GTG​AGT​GCA​TGG​ACT​GTG​GT

### Cell proliferation assay

Following a 24-hour transfection with siBIRC2 and siSEH1L, chondrocytes were seeded into a 96-well plate at a density of 3,000 cells per well. Subsequently, 10 μL of cell counting kit-8 (CCK-8) solution (DOJINDO, Japan) was added to each well at 24 and 48 h. The optical density (OD) value for each well was measured at 450 nm after a 2-h incubation at 37°C using a microplate reader (BioTek, United States).

### Statistical analysis

The data were expressed as the mean ± standard deviation (SD) as shown. For statistical analysis, differences between groups were assessed using one-way ANOVA after verifying the homogeneity of variance, while data within the same group were analyzed using Student’s t-test. A two-way analysis of variance (ANOVA) with Sidak’s test was employed for conducting multiple comparisons. Sidak’s multiple comparisons test was utilized to examine gene correlations. The expression levels of genes in both HC and OA groups were compared using the Wilcoxon rank-sum test. All experiments were conducted for at least three times. A *P*-value of less than 0.05 was deemed statistically significant.

## Results

### Patients

**FIGURE 1:**
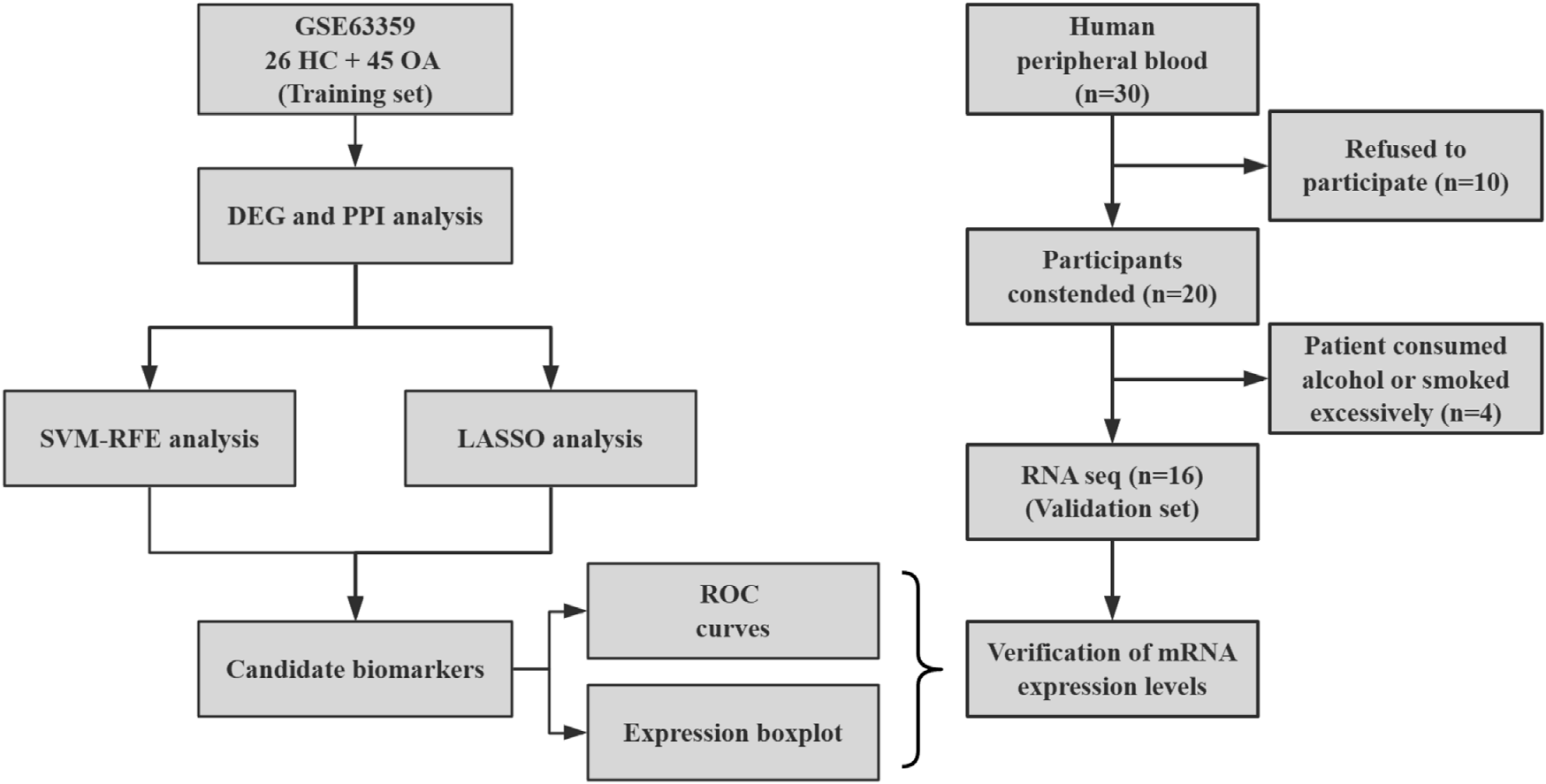
Flow chart of study analysis.

A total of 30 individuals were initially screened for eligibility to participate in the study. Out of these, 10 individuals declined to participate after being informed of the study’s details. Consequently, 20 participants who provided written informed consent were enrolled in the study. However, during the course of the study, 4 participants withdrew due to excessive alcohol consumption or smoking habits to maintain consistency with the control group. Ultimately, the final cohort consisted of 11 patients (2 males and 9 females, aged between 55 and 70 years) and 5 healthy volunteers, all of whom successfully completed the study and were subsequently included in the statistical analysis (Figure 1).

### DEGs and PPI in the training set

**FIGURE 2:**
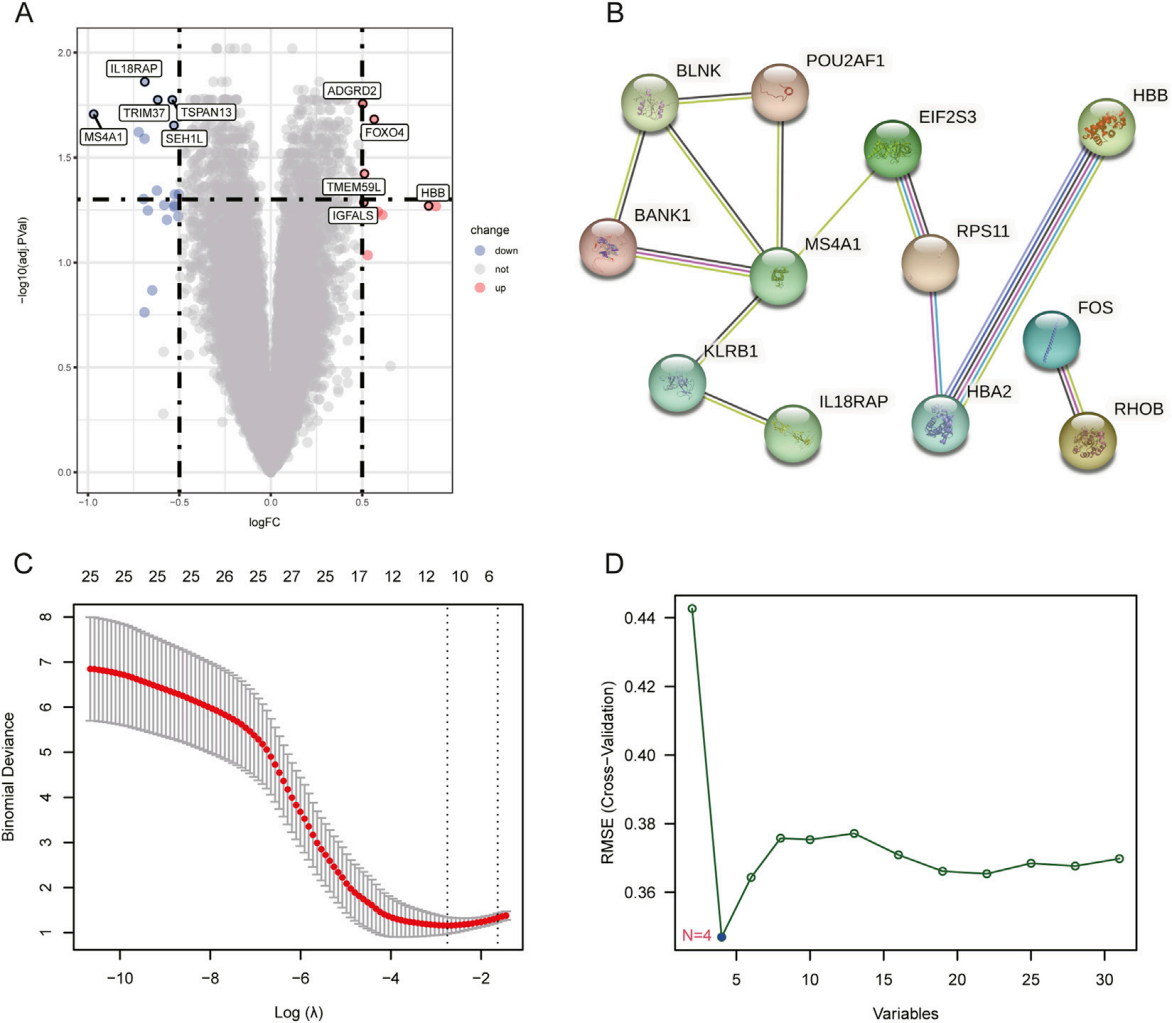
DEGs and PPI in the training set. **(A)** Volcano plot of DEGs between normal and OA samples. The top five upregulated and downregulated DEGs are indicated. **(B)** PPI network of DEGs. Different edge colors represent different types of associations. **(C)** Candidate biomarkers filtered out by the LASSO method. The optimal lambda value represented by the dashed line minimizes cross-validated error. **(D)** Candidate biomarkers identified by the SVM-RFE algorithm to find the feature set with the lowest RMSE. RMSE: Root Mean Squared Error.

A total of 31 genes were finally analyzed to be DEGs between healthy donors and OA patients in the training set. Among all, 21 DEGs were shown to be downregulated while the left ten were upregulated in OA patients. The first five downregulated DEGs with the smallest *P*-values were *IL18RAP* (interleukin 18 receptor accessory protein), *TRIM37* (tripartite motif containing 37), *TSPAN13* (tetraspanin 13), *MS4A1* (membrane spanning 4-domains A1), and *SEH1L* (SEH1 like nucleoporin), whereas the first five upregulated DEGs were *ADGRD2* (adhesion G protein-coupled receptor D2), *FOXO4* (forkhead box O4), *TMEM59L* (transmembrane protein 59 like), *IGFALS* (insulin like growth factor binding protein acid labile subunit), and *HBB* (hemoglobin subunit beta) (Figure 2A). According to the PPI network, MS4A1 had the strongest correlation with other proteins including POU2AF1 (POU class 2 homeobox associating factor 1), BLNK (B cell linker), BANK1 (B cell scaffold protein with ankyrin repeats 1), EIF2S3 (eukaryotic translation initiation factor 2 subunit gamma), and KLRB1 (killer cell lectin like receptor B1) (Figure 2B). The LASSO logistic regression model identified a model consisting of two genes, namely, *IL18RAP* and *TSPAN13*, could obtain the lambda.1se (Figure 2C). The SVM-RFE algorithm discovered the combination of *SEH1L*, *CAMP* (cathelicidin antimicrobial peptide), *IL18RAP*, and *BIRC2* (baculoviral IAP repeat containing 2) could achieve the lowest RMSE (Figure 2D). These five genes were then considered to be potential candidate biomarkers.

### Development of candidate biomarkers

**FIGURE 3:**
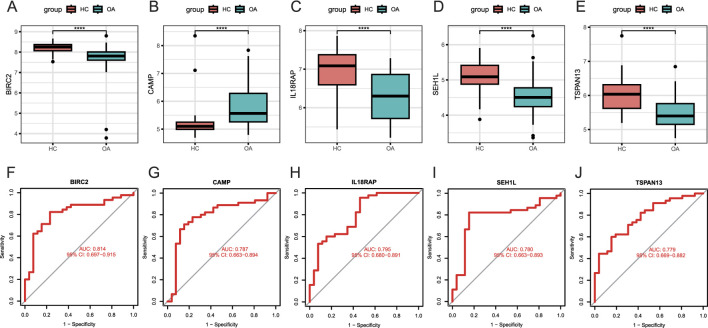
Development of candidate biomarkers. **(A–E)** Expression levels of BIRC2, CAMP, IL18RAP, SEH1L, and TSPAN13 from the training set. The *y*-axis indicates the expression level. **(F–J)** ROC curves of BIRC2, CAMP, IL18RAP, SEH1L, and TSPAN13. The *x*-axis indicates false positive rate, calculated by 1-specificity, while the *y*-axis indicates the specificity. HC: healthy control (n = 26). OA: osteoarthritis (n = 45). *P < 0.05; **P < 0.01; ***P < 0.001; ****P < 0.0001.

We then checked the peripheral expression levels of the aforementioned five candidate biomarkers in the training set and found that healthy donors had significantly higher circulating levels of BIRC2, IL18RAP, SEH1L, and TSPAN13 but a significantly lower level of CAMP, compared to the OA patients (Figures 3A–E). Notably, BIRC2 had the best ability to identify healthy people from OA patients, with an AUC of 0.814 (95% CI: 0.697-0.915) (Figure 3F). The AUCs for the remaining four candidate biomarkers did not reach 0.8; specifically, the AUC was 0.787 (95% CI: 0.663-0.894) for CAMP, 0.795 (95% CI: 0.680-0.891) for IL18RAP, 0.780 (95% CI: 0.663-0.893) for SEH1L, and 0.779 (95% CI: 0.669-0.882) for TSPAN13 (Figures 3G–J). Taken together, BIRC2 had a very good discriminatory effect among the five candidate biomarkers, followed by IL18RAP, CAMP, SEH1L, and TSPAN13 had a relatively poor performance in the training set.

### Verification of candidate biomarkers

**FIGURE 4:**
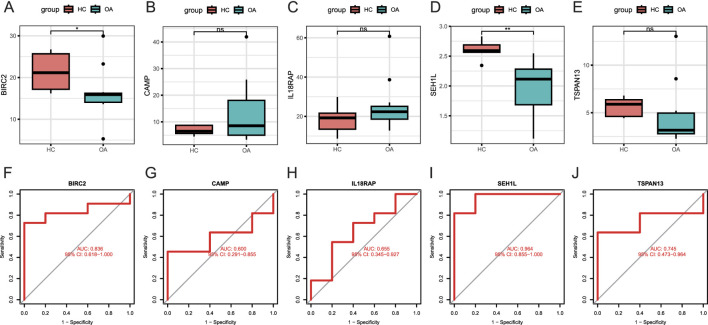
Verification of candidate biomarkers. **(A–E)** Expression levels of BIRC2, CAMP, IL18RAP, SEH1L, and TSPAN13 in peripheral blood samples from the validation set by RNA-seq assay. The *y*-axis indicates the expression level. **(F–J)** ROC curves of BIRC2, CAMP, IL18RAP, SEH1L, and TSPAN13. The *x*-axis indicates false positive rate, calculated by 1-specificity, while the *y*-axis indicates the specificity. HC: healthy control (n = 5). OA: osteoarthritis (n = 11). *P < 0.05; **P < 0.01; ***P < 0.001.

We next examined the peripheral expression levels of the five candidate biomarkers described above in our validation set. Results showed that the levels of BIRC2 (Figure 4A) and SEH1L (Figure 4D) were remarkably higher in healthy donors than OA patients, consistent with the results of the training set from the public database. TSPAN13 (Figure 4E) also demonstrated a higher level in healthy donors, but was not significantly. The circulating levels of CAMP (Figure 4B) and IL18RAP (Figure 4C) were similar in both groups. Although the AUCs of all five markers were greater than 0.6, their efficacy varied considerably. SEH1L owned the largest AUC of 0.964 (95% CI: 0.855-1.000) (Figure 4I), even better than that in the training set. BIRC2 also displayed a larger AUC of 0.836 (95% CI: 0.618-1.000) (Figure 4F) in the training set. The AUCs for the other three biomarkers, though all greater than 0.5, were not as good as those in the training set (Figures 4G, H, J). Therefore, it was indicated that both BIRC2 and SEH1L had the capacity to differentiate healthy people from OA patients at the peripheral level.

### Knockdown of *BIRC2* and *SEH1L* inhibits chondrocyte proliferation

**FIGURE 5:**
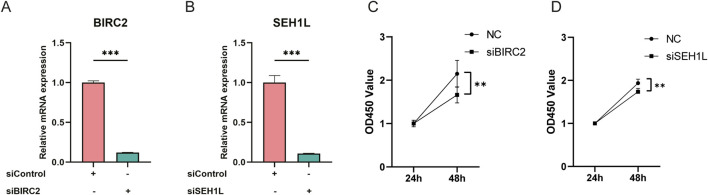
Knockdown of *BIRC2* and *SEH1L* inhibits human chondrocyte proliferation. **(A)** Verification of *BIRC2* knockdown in human chondrocytes by qPCR assay. **(B)** Verification of *SEH1L* knockdown in human chondrocytes by qPCR assay. **(C)** Knockdown of *BIRC2* inhibited chondrocyte proliferation. **(D)** Knockdown of *SEH1L* inhibited chondrocyte proliferation. *P < 0.05; **P < 0.01; ***P < 0.001.

To investigate the impact of *BIRC2* and *SEH1L* on chondrocyte function, we employed siRNA to specifically down-regulate the expression of these genes in human chondrocytes, which was then validated by qPCR assay to ensure the knockdown efficacy compared to the siControl group (Figures 5A, B). At the 48-hour after incubation, we noted a significant reduction in cell proliferation within the knockdown groups targeting *BIRC2* and *SEH1L* (Figures 5C, D). These findings suggest that the downregulation of *BIRC2* and *SEH1L* has a detrimental effect on chondrocyte proliferation, indicating their potential roles in the regulation of chondrocyte growth and maintenance.

## Discussion

OA is a significant global health issue that causes severe pain, disability, and economic hardship for patients, placing a substantial burden on public health (Pereira et al., 2011). A number of biomarkers have been studied for the diagnosis of OA (Liu et al., 2023). These biomarkers include proteins in joint fluid, inflammatory factors, and molecules associated with cartilage metabolism. For example, specific proteins based on serum or joint fluid (e.g., collagen degradation products, MMPs, etc*.*) can help assess the degree of joint damage and inflammatory state. However, no biomarkers have yet been widely accepted as a standard diagnostic tool for OA, and a combination of clinical symptoms and imaging is usually required for a comprehensive assessment.

Therefore, in this study we integrated bioinformatics analysis with machine learning to explore candidate biomarkers and assessed their diagnostic effectiveness through the use of ROC analysis. We demonstrated that both BIRC2 and SEH1L had the capacity to differentiate healthy people from OA patients at the peripheral level. Since the samples in this study were taken from peripheral blood, we can estimate the onset of knee OA by analyzing the expression of BIRC2 and SEH1L in their peripheral blood. Additionally, functional experiments were conducted to investigate the role of BIRC2 and SEH1L on human chondrocytes. Knockdown of *BIRC2* and *SEH1L* was achieved through siRNA, which was found to hold back chondrocyte proliferation.

BIRC2 is classified as a member of the anti-apoptotic protein family, which serves to inhibit apoptosis and facilitate cellular proliferation through its interactions with TRAF1 and TRAF2. This protein is related to BIRC5, which has recently been identified as a novel biological marker for cartilage stem/progenitor cells (CSPC) (Yuan et al., 2020). Supporting this notion, prior studies have demonstrated that mesenchymal stem cells derived from the umbilical cord Wharton’s jelly in younger women who undergo natural childbirth exhibit elevated expression levels of BIRC2, leading to the hypothesis that these cells possess enhanced stem cell functionality (Gil-Kulik et al., 2020). The researchers discovered that the knockdown of BIRC2 markedly compromised the cellular functions of CSPC (Zhang et al., 2024). The dysfunction of CSPC adversely affected the inherent repair capabilities of cartilage and disrupted cartilage homeostasis in OA. They propose that the candidate gene *BIRC2* is crucial for the maintenance of CSPC function, operating through the transcriptional co-activator YAP (Zhang et al., 2024). Therefore, we believe that BIRC2 plays a crucial role in knee OA patients.

SEH1L, a component of the nuclear pore complex (NPC), plays a critical role in the regulation of mTORC1 by acting as an inhibitor of the Rag GTPases (Bar-Peled et al., 2013). Ye et al. conducted a bioinformatics analysis that demonstrated a cooperative regulatory relationship among SEH1L, GRB2, and L-arginine in the modulation of the mTOR signaling pathway in OA (Yuan et al., 2024). The authors posit that SEH1L serves as a crucial gene associated with ubiquitination processes that contribute to immune infiltration in OA patients (Yuan et al., 2024). Their findings complement our conclusion that SEH1L holds promise as a biomarker for predicting the progression of OA, thereby enhancing the understanding of its role in the disease’s immune-related mechanisms. Furthermore, research has indicated that SEH1L is involved in the regulation of cell division through its influence on chromosome alignment and segregation (Platani et al., 2009). Recently, multiple studies have suggested that SEH1L may serve as a potential biomarker in cancer. Researchers demonstrated that SEH1L is downregulated in ovarian cancer and may act as a prognostic biomarker (J et al., 2021). The researchers additionally observed that SEH1L was significantly upregulated in hepatocellular carcinoma (HCC) (Feng et al., 2024). Furthermore, next-generation sequencing results indicated that SEH1L may play a role in the regulation of ferroptosis. Notably, the knockdown of SEH1L markedly induced ferroptosis and impeded the progression of HCC (Feng et al., 2024). Ferroptosis could also play a role in the pathogenesis of OA by regulating chondrocytes (Miao et al., 2022; Wang et al., 2022). It could be speculated that SEH1L affects the progression of OA by modulating the process of ferroptosis in chondrocytes.

The identification of two novel biomarkers for OA in peripheral blood offers substantial potential for improving diagnostic precision and formulating targeted therapeutic approaches. The presence of BIRC2 and SEH1L can significantly enhance the ability to diagnose OA at an early stage, thereby enabling timely clinical interventions that may mitigate disease progression and improve patient outcomes. Furthermore, the consistent monitoring of these biomarkers may provide valuable insights into the trajectory of the disease, allowing healthcare professionals to evaluate the effectiveness of ongoing treatments and make necessary adjustments based on the patient’s response. From a therapeutic perspective, a comprehensive understanding of the newly identified biomarkers and their roles in the pathophysiological processes of OA can inform the development of targeted therapies. Overall, the integration of novel biomarkers into clinical practice has the potential to transform the management of OA, enhancing both diagnostic and therapeutic strategies.

Although our findings show that BIRC2 and SEH1L are significantly downregulated in the peripheral blood of patients with knee osteoarthritis, and can definitely affect the proliferation of chondrocytes *in vitro*, which has important clinical application potential, this study still has its limitations. In Liu’s research (Liu, 2019), the author utilized datasets derived from synovial tissue to conduct a comparative analysis. The findings indicated that BIRC2 exhibited significant differential expression when comparing rheumatoid arthritis (RA) samples to normal controls (NC). However, this significant difference was not observed in the comparison between OA samples and NC in their research. This discrepancy in findings may be attributed to variations in sample sources, which can influence the expression profiles of specific genes and ultimately affect the conclusions drawn regarding their relevance in different pathological contexts. Besides, we recognize the sample size limitations in our study, as well as the variability in disease stages. Additionally, we observe that the datasets predominantly comprise samples from specific populations, which may limit the generalizability of our results to other demographic groups. Expanding the clinical sample size may make our results more persuasive.

## Conclusion

Utilizing bioinformatics analyses and machine learning methodologies, we systematically identified two novel biomarkers, BIRC2 and SEH1L, which might function as potential instruments for clinical diagnosis and therapeutic interventions in knee OA. Knockdown of these two genes could significantly repress human chondrocyte proliferation *in vitro*.

## Data Availability

The datasets presented in this study can be found in online repositories. The names of the repository and accession numbers can be found in the article.
